# Reply to Deora et al. Multiplexing for *Plasmodium* spp.? Think Again! Comment on “Bhowmick et al. Dry Post Wintertime Mass Surveillance Unearths a Huge Burden of *P. vivax*, and Mixed Infection with *P. vivax* *P. falciparum*, a Threat to Malaria Elimination, in Dhalai, Tripura, India. *Pathogens* 2021, *10*, 1259”

**DOI:** 10.3390/pathogens11080832

**Published:** 2022-07-26

**Authors:** Ipsita Pal-Bhowmick, Tulika Nirmolia, Apoorva Pandey, Sarala K. Subbarao, Aatreyee Nath, Susmita Senapati, Debabrata Tripathy, Rocky Pebam, Suman Nag, Rajashree Roy, Dipanjan Dasgupta, Jayanta Debnath, Kongkona Gogoi, Karuna Gogoi, Lakhyajit Borah, Rajdeep Chanda, Arup Borgohain, Chelapro Mog, Ujjwal Sarkar, Phiroz Gogoi, Bishal Debnath, Jyotish Debbarma, Dibya Ranjan Bhattacharya, Pyare Lal Joshi, Harpreet Kaur, Kanwar Narain

**Affiliations:** 1 Regional Medical Research Center-Northeast Region (RMRC-NE)-ICMR, Dibrugarh 786001, India; tulikanirmolia@gmail.com (T.N.); senapatitiku999@gmail.com (S.S.); debabrata.tripathy07@gmail.com (D.T.); snag8540@gmail.com (S.N.); royrajashree6@gmail.com (R.R.); dipanjandasgupta89@gmail.com (D.D.); debnathjayantabsc@gmail.com (J.D.); koko1.gogoi@gmail.com (K.G.); karunarmrc@gmail.com (K.G.); lakhyaborah01@gmail.com (L.B.); chelapromog348@gmail.com (C.M.); ujjwalsarkar188@gmail.com (U.S.); phirozgogoi@gmail.com (P.G.); bishaldebnath89@gmail.com (B.D.); jd45683968@gmail.com (J.D.); drbhattacharyya@yahoo.com (D.R.B.); kanwar_narain@hotmail.com (K.N.); 2 Indian Council of Medical Research (ICMR), Ramalingaswami Bhavan, Delhi 110029, India; apoorva.icmr@gmail.com (A.P.); kaurh.hq@icmr.gov.in (H.K.); 3 Formerly National Institute of Malaria Research-ICMR, Delhi 110077, India; subbaraosk@gmail.com; 4 Northeastern Space Applications Centre, Department of Space, Government of India, Umiam 793103, India; aatreyeen04@gmail.com (A.N.); rocky.pebam@gmail.com (R.P.); arupborgohain@gmail.com (A.B.); 5 Mizoram University, Aizawl 796004, India; rajdeepchanda1991@gmail.com; 6 Formerly National Vector Borne Disease Control Program (NVBDCP), Delhi 110054, India; doctorjoshi00@gmail.com

We thank Deora et al. [1] for their attention to our work; however, we would like to mention the following:

The semi-nested multiplex method that we used has also been used by other researchers such as refs. [2,3,4], and we cited Siwal et al. in the methods section of our paper. 

Siwal et al. [2] found considerably high proportions of mixed infection of 13% and Haidi et al. [3] found 1.9%, respectively.

In our paper, using the nested multiplex PCR method, we reported considerable numbers (26) of mixed infection cases, comprising 36.11% of the total cases. Among them, there are three mixed infection cases, in which one of the species is sub-microscopic and the other one is microscopically detectable, i.e. with higher density. This shows that low-density species could be detected in presence of other higher-density species by this nested multiplex PCR method.

While Siwal et al. and Haidi et al. [2,3] have used dry blood spots, we used whole blood, which can further increase the sensitivity to detect the lower density infections. In our experience, the method of sample collection can significantly impact the sensitivity of parasite speciation, particularly in cases of low-density parasitaemia. We also mentioned this in the paper, distinguishing our study from a previous one in this regard.

We would like to mention that we checked a subset of 15 samples from our study which were identified as either *P. falciparum or P. vivax* by multiplex PCR method. We obtained similar results (9 *P. falciparum* and 6 *P. vivax*) even when nested PCR was performed individually for the two species. No mixed infection was detected in these samples. We would also like to add that in another subset of 25 samples, collected from different study area and durations, the results were similar to multiplex PCR.A manuscript on comparative study between these two methods is already under preparation with a larger set of samples.

Deora et al. quoted Snounou and Singh’s book chapter [5] and Snounou et al. [6] stating that, “some sensitivity will invariably be lost as a result of competition between the different amplified fragments for the limited materials present in the reaction” and mentioning that “if a species is present at a burden 10^2^–10^4^ times lesser than the other species in a sample, it could not be detected in a multiplex PCR Nest 2 reaction”. While this study was conducted on the culture blood mixed with infected chimpanzee blood, our study utilized whole patient blood to test the presence of *P. falciparum* and *P. vivax*. We thus believe that in case of mixed infection, the efficiency of PCR to detect these two species can be dependent on the sample types. A manuscript on comparative studies for different sample types is under preparation.

The other two references [7,8] cited by the commenting authors used different methods and primer sets and one of them used a gene target different from ours. The type of blood specimens was also not mentioned. Hence, the findings of these studies are not necessarily comparable and applicable to us. The differences in primers and targets can vary the results, and this has been pointed out by their quoted paper itself [5]. The same is elaborated in the subsequent points.

Deora et al. suggested that “multiplexing is associated with a loss of sensitivity and is unacceptable in low transmission and low endemic settings because it may fail to reveal the true proportional burden of different *Plasmodium* species in areas with low parasite loads even for one of the investigated species”. In this context, firstly we would like to emphasize that we can term our area neither as low-endemic nor as low-transmission for any of the species. We found submicroscopic levels of one species in presence of microscopic density of the other from our study area. Additionally, as shown in the subset of samples tested, these results are comparable with that of the multiplex PCR.

Secondly, our aim was to point out the hidden burden using a low-cost PCR technique. Hence, nowhere in our paper did we claim that the obtained proportion or proportional burden is the absolute one. One can unearth even greater burden of any infection by employing different and more sensitive methods, which may alter the proportional burden. We observed the same using a more sensitive qPCR technique. 

“The loss of sensitivity in a multiplexed reaction stems from the intrinsic property of the primers” as mentioned by Deora et al., does not necessarily hold true. In this context, other researchers have used different primer sets which target the ssrRNA gene of the *Plasmodium* parasites employing the semi-nested multiplex PCR assays to detect the parasites. Snounou et al. [5] (the paper referred by Deora et al., in order to drive home the point of non-utility of multiplex PCR for mixed detection)also discussed these studies and mentioned that their inability to detect low-density infection can be “primer-specific”. Describing a rapid, single-round multiplex PCR assay, Padley et al. prepared a mixture of *P. vivax:P. falciparum* into four ratios of 1:1.4, 1:0.14, 1:0.014, and 1:0.0014 and they detected both the species in all four dilutions [9].

Most importantly, in our paper, we never claimed that we are reporting the ‘real/absolute burden’ for the mixed infections. Rather, we emphasized that even with the routine techniques of RDT, microscopy, and other currently deployed methods in the field, we are still missing out on a huge burden of infections. Particularly, the cases of *P. vivax* in a reportedly *P. falciparum*-dominated area and mixed infections should be further investigated for the presence of *P. vivax*. 

More sensitive molecular detection methods can reveal a higher burden of low-density infections, single or mixed. The key takeaway of our paper was, to reiterate, a burden of *P. vivax* and mixed infection being missed by the routine procedures exists and it can be unearthed by the simple, cost-effective normal, multiplex PCR technique involving only finger-prick blood. Even the chapter of *Snounou and Singh* [10] cited by the authors states that “Multiplexing” is nonetheless an excellent cost-saving strategy, provided that the loss of sensitivity, whose extent can be established experimentally, falls within the tolerance of each particular investigation”. Our investigation, particularly with the whole blood patient samples, has been able to unearth a vast hidden burden of *P. vivax* and mixed infections in a cost-effective manner. Deora et al. suggested that the sensitivity to detect mixed infections can be less, but we nowhere claimed that this detection technique is the ultimate and most sensitive technique or the best technique for mixed infection detection. Rather, we would like to emphasize that with this technique itself; in a low-cost manner, we were able to detect a vast proportion of *P. vivax* and mixed cases to prove the point that a huge burden of *P. vivax* and mixed-case burden exists beyond the RDT and microscopic detection limit.

In conclusion, it is evident that the sensitivity to detect mixed species can vary depending on each of the factors like the targeted gene set, primers used, sample type and the preservation, extraction technique, and the combination of these factors as well, as unique for each specific experiment. In our paper, we unveiled a hitherto hidden burden of *P. vivax* and mixed infections in the study area using a cost-effective molecular method. 
